# Modelling the factor structure of the Child Depression Inventory in a population of apparently healthy adolescents in Nigeria

**DOI:** 10.1371/journal.pone.0193699

**Published:** 2018-03-09

**Authors:** Samson Bamidele Olorunju, Onoja Matthew Akpa, Rotimi Felix Afolabi

**Affiliations:** Department of Epidemiology and Medical Statistics, College of Medicine, University of Ibadan, Ibadan, Nigeria; Universita degli Studi di Pisa, ITALY

## Abstract

**Background:**

Childhood and adolescent depression is common and often persists into adulthood with negative implications for school performances, peer relationship and behavioural functioning. The Child Depression Inventory (CDI) has been used to assess depression among adolescents in many countries including Nigeria but it is uncertain if the theoretical structure of CDI appropriately fits the experiences of adolescents in Nigeria. This study assessed varying theoretical modelling structure of the CDI in a population of apparently healthy adolescents in Benue state, Nigeria.

**Methods:**

Data was extracted on CDI scale and demographic information from a total of 1, 963 adolescents (aged 10–19 years), who participated in a state wide study assessing adolescent psychosocial functioning. In addition to descriptive statistics and reliability tests, Exploratory Factor Analysis (EFA) and Confirmatory Factor analysis (CFA) were used to model the underlying factor structure and its adequacy. The suggested new model was compared with existing CDI models as well as the CDI’s original theoretical model. A model is considered better, if it has minimum Root Mean Square Error of Approximation (RMSEA<0.05), Minimum value of Discrepancy (CMIN/DF<3.0) and Akaike information criteria. All analyses were performed at 95% confidence level, using the version 21 of AMOS and the R software.

**Results:**

Participants were 14.7±2.1 years and mostly male (54.3%), from Monogamous homes (67.9%) and lived in urban areas (52.2%). The measure of the overall internal consistency of the 2-factor CDI was α = 0.84. The 2-factor model had the minimum RMSEA (0.044), CMIN/DF (2.87) and least AIC (1037.996) compared to the other five CDI models.

**Conclusion:**

The child depression inventory has a 2-factor structure in a non-clinical general population of adolescents in Nigeria. Future use of the CDI in related setting may consider the 2-factor model.

## Introduction

Globally, one of the major contributors to the burden of diseases is depression and it has been shown to be the leading cause of disability in terms of total years lost [1]. Depressive symptoms do not only start at a young age, often they extend to adulthood; they are more intense and difficult to manage than normal sadness feelings [1–2]. Studies assessing depression in different population settings in Nigeria have been published [3–8]. In a recent study conducted among university undergraduate students in Nigeria, prevalence of severe depression was put at 7% [4] while Amoran et al. [6] showed that depression was more common in rural (7.3%) than urban (4.2%) centres.

Prior to 1960, little or close to nothing was mentioned about childhood depression in the literature but in the past five decades, existence of childhood depression is now widely recognised [9–11]. Consequently, assessment tools have been developed over the years to assess the nature of childhood depression. Some of the scales include the Centre for Epidemiological Study Depression Scale for Children (CES-DC) [12], The Children’s Depressive Rating Scale (CDRS) [13], the Children’s Depression Scale (CDS) [14], the Reynold’s Child Depression Scale (RCDS) [15] and the Child Depression Inventory (CDI) [16].

However, the CDI has been cited in the literature as one of the most viable instruments for assessing depressive symptoms both in children and young adults. The CDI, a downward extension of the BDI, consists of 27 items assessing depressive symptoms. Most of these items on the CDI are derivations of the BDI with some word changes [17]. It was initially designed as a means of distinguishing youths with psychiatric diagnoses of major depressive symptoms from “normal” schoolchildren [18]. A 10-item version of the CDI; Children’s Depression Inventory—Short Form (CDI-S) has also been published [19, 20]. Just as the original CDI, the CDI-S was designed to be used in children and adolescents as young as 7 years old and its psychometric properties have been reported in previous studies [21,22].

Varying factor models and versions have been suggested for the structure of the CDI in the literature [23–26]. For instance, Kovacs [16] in the original model, proposed a five-factor and a single second-order factor. The factors proposed are Anhedonia, Negative self-esteem, Ineffectiveness, Interpersonal problems and Negative mood. However, Craighead et al. [24] proposed a six-factor model that has been more widely reported [25–27]. Their model identified factors like School Problems, Social Problems, Self-Depreciation, Dysphoria, Externalizing, and Biological Dysregulation. Among a population of Asian adolescents, a three-factor structure was reported [23]. Despite its wide usage and assessment of factor structure, Weiss et al. [17] opined that the analytic techniques used may limit our knowledge of the internal structure of the CDI. Specifically, the method of factor extraction or rotation, the population being studied and other methodological limitations could be the reason for these limited knowledge of the internal structure of the CDI [17, 23].

The Child Depression Inventory has been used to assess prevalence of depression among adolescents in Nigeria but no study has investigated the factor structure of the original CDI model in Nigeria. It is therefore uncertain if the theoretical structure of the CDI appropriately fits the experiences of adolescents in Nigeria. In addition to rudimentary psychometric properties, this study compared six models for the theoretical structure of the CDI in a population of apparently healthy adolescents in Benue state, Nigeria. We hypothesized that the experiences of Adolescents in the Nigerian setting does not completely follow the original 5-factor model of the CDI.

## Methods

### Data extraction and instruments

The data used for this study were extracted from 1,963 participants in a cross-sectional survey database. Participants were consenting, school attending Adolescents, aged 10–19 years who filled a self-administered questionnaire. These participants were drawn from purposely selected (based on size, sex composition and from each senatorial zone) secondary schools across Benue state, Nigeria. Benue State is in the north central (middle belt region) of Nigeria, and typically represents a good strata of Nigerian adolescents. Participants in the parent study were secondary school students who could read, write and understand English language well. They were also capable of understanding and providing responses to the questionnaire items. Those who had issues understanding any particular question indicated and were promptly attended to in a manner that will not suggest a response to them. Further details on the sampling techniques, are described in Akpa, Bamgboye and Baiyewu [28].

### Study instruments

The study instrument consisted of a section on socio-demographic information including age, sex and related family characteristics. The questionnaire also consisted the 27-item CDI measured on a 3-point Likert scale, where 2 indicated definite symptoms, 1 indicated mild symptoms, and 0, absence of symptoms. The total score ranged from 0 to 54, with higher scores representing severe depressive symptomatology [18, 28].

### Data management, descriptive analysis and reliability of the CDI

Extracted data were assessed for outliers and consistency in response. Participant age was grouped into early-adolescence (<13 years old), mid-adolescence (13-17years) and late-Adolescence (18-19years). The socio-demographic characteristics of the adolescents and proportion responding to each item of the CDI were summarised using descriptive statistics. Cronbach alpha (α) and polychoric/Ordinal Alpha (α_p_) (because of the ordinal nature of the response scale) were used to assess the reliability of the CDI models.

### Statistical models

A two–step approach (exploratory and confirmatory) was used in the factor analysis. The samples were randomly divided into two and the smaller sample (n = 980) was used for exploratory factor analysis (EFA), while the larger sample (n = 983) was used for the confirmatory factor analysis (CFA). Both the *Kaiser-Meyer-Olkin* (KMO) measure of sampling adequacy and the Bartlett’s test of sphericity were used to test the adequacy of the sample for the factor analysis.

**Fig 1:**
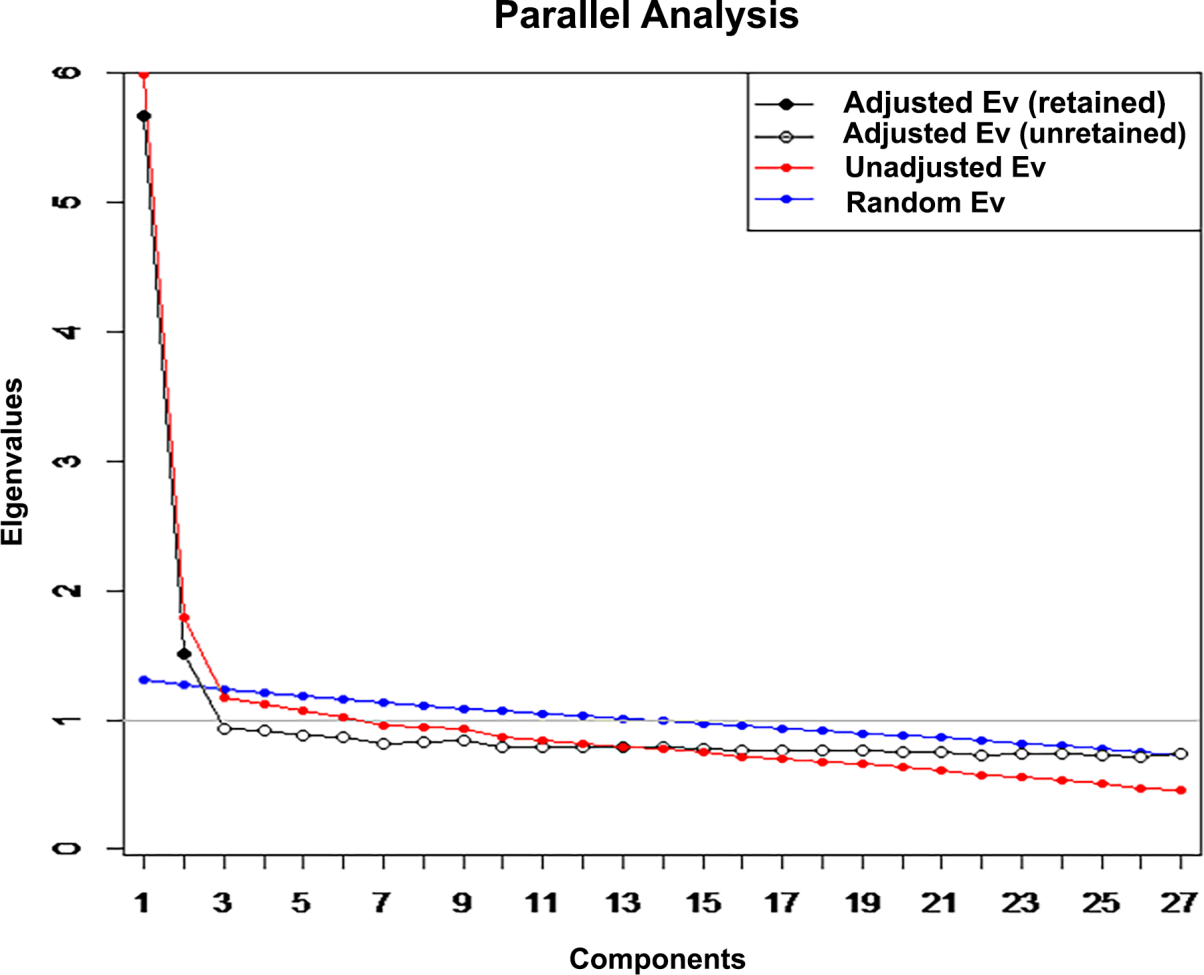
Scree plot showing the number of factors retained in the CDI.

In the EFA, the number of factors to retain and the items loaded on each factor were determined using multiple strategies. The Horn’s parallel analysis (Fig 1) (using R statistical software) [29,30], eigen values >1 and the scree plot were used to determine the number of factors to retain. Items are said to load on a factor where they have a factor loading (in absolute value) ≥0.4 and it is the highest as compared to other factors.

Three literature based theoretical models, Model I: Six-Factor Model [24]; Model II: Five-Factor Model [16]; Model III: Three Factor Model [23, 31] and the new two-factor model (Model IV) extracted from the data used for the EFA were comparatively assessed in a confirmatory factor analysis. Using the recommended cutoff of 20 (Kovacs), the participants were categorised as depressed or normal and the two factor model was tested on these two samples respectively called Model V and Model VI. The CFA were carried out using the Analysis of Moment Structure (AMOS) software version 21. Fit indices such as the Root Mean Square Error of approximation (RMSEA), the Comparative fit index (CFI), Goodness of Fit index (GFI), Minimum discrepancy (CMIN), the minimum discrepancy divided by degrees of freedom (CMIN/DF), Root Mean Square Residual (RMR), Adjusted goodness of fit index (AGFI) and Parsimony Goodness of Fit Index (PGFI) were used to check the adequacy of the models while the Akaike Information Criteria (AIC), and the Bayesian Information Criteria (BIC) were used for model comparison. A model with RMSEA ≤0.05, GFI≥ 0.9, CFI≥ 0.9, and lowest AICs/BICs is adjudged best.

### Ethics approval

Ethical approval for the parent study was obtained from the University of Ibadan/University College Hospital (UI/UCH) Ethics Committee (with the ethics approval number UI/EC/12/02350 and Benue state Ministry of Health’s ethics committee (with the reference number MED/261/VOL.1/56). We obtained verbal informed consent from the caretakers, or guardians on behalf of the students who must provide ascent before enrolment into the study. Participants were free to withdraw from the study at any time without suffering any consequences.

## Results

### Descriptive statistics

Participants were mostly male (54.0%) while 46.0% of them were female. Also, majority (63.0%) of the participants were in their mid-adolescence (13-17years) while 27.6% and 9.5% were in their early adolescence (<13years) and late adolescence (18-19years) respectively. Most participants (52.2%) are living in urban areas while 47.8% reported to be residing in the rural areas. Most adolescents (74.8%) came from homes where parents are living together (Table 1).

**Table 1:** Socio demographic characteristics of respondents.

Variable	Frequency	Percentage (%)
**Gender**		
*Male*	1065	54.3
*Female*	895	45.7
**Age**		
**Mean age(SD)**	**14.71**	**2.05**
*Early Adolescents(<13years)*	541	27.6
*Mid-adolescents(13-17years)*	1236	63.0
*Late Adolescents(18-19years)*	186	9.5
**Religion**		
*Christianity*	1880	96.3
*Islam*	69	3.5
*Others*	3	0.2
**Tribe**		
*TIV*	1124	57.8
*Idoma*	142	7.3
*Igede*	375	19.3
*Others*	304	15.6
**Family Type**		
*Monogamy*	1289	67.9
*Polygamy*	608	32.1
**Area of Residence**		
*Rural Area*	882	47.8
*Urban Area*	965	52.2
**Family Status**		
*Parents are together*	1427	74.8
*Parents are divorced*	81	4.2
*Parents live apart*	136	7.1
*Single parent*	265	13.9
**Father's highest level of education**		
*No formal education*	225	11.9
*Primary*	227	12.0
*Secondary*	447	23.7
*Tertiary*	704	37.2
*Others*	287	15.2
**Father's occupation**		
*Farming*	641	33.4
*Trading*	149	7.8
*Civil servant*	742	38.7
*Employee of private organisation*	144	7.5
*Others*	242	12.6
**Mother's highest level of education**		
*No formal education*	289	15.3
*Primary*	367	19.5
*Secondary*	486	25.8
*Tertiary*	521	27.6
*Others*	222	11.8
**Mother's occupation**		
*Farming*	619	32.5
*Trading*	532	27.9
*Civil servant*	442	23.2
*Employee of private organisation*	126	6.6
*Others*	188	9.9

### Item level responses and internal consistency of measures

The proportion of respondents endorsing “always” for having the depressive feelings as measured by the items of the CDI were generally low (Table 2). Precisely, they were less than 10% of the total responses per item. However, some items like things bother me all the time (10.5%), I can never be as good as other kids (13.5%), and I never have fun at school (13.3%) had proportions above 10% that always felt the symptoms described. Some of the sub-scales of the original theoretical model of the CDI had poor reliability. For instance, while the Anhedonia subscale had a Cronbach alpha of 0.36, the Ineffective sub-scale’s reliability was 0.41 (Table 3). The reliability of the subscales of the two-factor structure of the CDI was high. For instance, factor 1 had a Cronbach Alpha of 0.87, factor 2 had a Cronbach Alpha of 0.55 while the overall Cronbach Alpha (Polichoric alpha) was 0.84 (0.89) (Table 4)

**Table 2:** Proportion responding to items on the CDI.

	Not at all	Sometimes	Always
I am sad all the time	45.6	47.9	6.5
I am sure that terrible things will happen to me	65.6	26.7	7.7
I feel like crying everyday	61.5	31.9	6.5
Things bother me all the time	36.7	52.8	10.5
I sleep pretty well	13.8	50.8	35.4
I am tired all the time	37.2	53.3	9.5
Most days I don't feel like eating	33.3	57.3	9.4
I don't worry about aches and pains	34.1	52.3	13.6
I get into fights all the time	72.7	20.6	6.7
Nothing will ever work for me	67.2	24.9	7.9
I like myself	9.5	27.2	63.3
All bad things are my fault	64.4	28.2	7.4
I want to kill myself	82.4	14.1	3.5
I look ugly	64.4	26.9	8.7
Nobody really loves me	58.6	32.1	9.3
I do everything wrong	62.2	33.2	4.6
Nothing is fun at all	45.9	45.0	9.1
I am bad all the time	69.7	26.6	3.7
I cannot make up my mind about things	29.9	60.3	9.8
Doing school work is not a big problem	24.7	44.5	30.8
I never have fun at school	44.2	42.6	13.3
My school work is alright	15.6	47.9	36.5
I can never be as good as other kids	51.9	34.6	13.5
I like being with people	11.2	43.4	45.4
I do not feel alone	25.6	53.3	21.1
I have plenty of friends	23.9	38.7	37.4
I never do what I am told	48.3	43.7	8.1

**Table 3:** Reliability of the Kovacs model of CDI instrument and its subscales.

				Scale Reliability	
Item codes	Subscales and item statement	Mean	SD	α	αp
	**Negative Self Esteem Scale**	**2.45**	**2.041**	**0.608**	**0.71**
CD03	I do everything wrong	0.42	0.580		
CD14	I look ugly	0.44	0.649		
CD25	Nobody really loves me	0.51	0.660		
CD24	I can never be as good as other kids	0.62	0.712		
CD07	I like myself	0.46	0.662		
	**Anhedonia Scale**	**2.84**	**1.622**	**0.356**	**0.44**
CD04	Nothing is fun at all	0.63	0.644		
CD12	I like being with people	0.66	0.671		
CD21	I never have fun at school	0.69	0.692		
CD22	I have plenty of friends	0.86	0.772		
	**Interpersonal Scale**	**1.71**	**1.642**	**0.617**	**0.78**
CD05	I am bad all the time	0.34	0.546		
CD08	All bad things are my fault	0.43	0.628		
CD26	I never do what I am told	0.60	0.634		
CD27	I get into fights all the time	0.34	0.60		
	**Ineffectiveness Scale**	**4.00**	**1.821**	**0.411**	**0.48**
CD15	Doing school work is not a big problem	0.94	0.743		
CD16	I sleep pretty well	0.78	0.667		
CD17	I am tired all the time	0.72	0.625		
CD18	Most days I don't feel like eating	0.76	0.608		
CD23	My school work is alright	0.79	0.691		
	**Negative Mood Scale**	**5.88**	**2.731**	**0.623**	**0.71**
CD01	I am sad all the time	0.61	0.608		
CD02	Nothing will ever work for me	0.41	0.631		
CD06	I am sure that terrible things will happen to me	0.42	0.63		
CD09	I want to kill myself	0.21	0.485		
CD10	I feel like crying everyday	0.45	0.615		
CD11	Things bother me all the time	0.74	0.636		
CD13	I cannot make up my mind about things	0.80	0.597		
CD19	I don't worry about aches and pains	1.21	0.659		
CD20	I do not feel alone	1.04	0.683		

SD_Standard deviation; α-Cronbach’s alpha; αp-Polychoric alpha

**Table 4:** Factor loadings of the 2-factor model of the CDI.

		Combined sample	
Item code	Item statement	Factor 1	Factor 2
CDI01	I am sad all the time	**0.553**	0.019
CDI06	I am sure that terrible things will happen to me	**0.609**	0.150
CDI10	I feel like crying everyday	**0.590**	0.130
CDI11	Things bother me all the time	**0.582**	0.009
CDI17	I am tired all the time	**0.534**	0.069
CDI18	Most days I don't feel like eating	**0.432**	-0.048
CDI27	I get into fights all the time	**0.562**	0.061
CDI02	Nothing will ever work for me	**0.585**	0.048
CDI08	All bad things are my fault	**0.541**	0.070
CDI09	I want to kill myself	**0.534**	0.210
CDI14	I look ugly	**0.524**	0.139
CDI25	Nobody really loves me	**0.560**	0.112
CDI03	I do everything wrong	**0.555**	0.094
CDI04	Nothing is fun at all	**0.505**	0.028
CDI05	I am bad all the time	**0.589**	0.094
CDI13	I cannot make up my mind about things	**0.434**	0.007
CDI21	I never have fun at school	**0.402**	-0.056
CD124	I can never be as good as other kids	**0.521**	0.140
CDI26	I never do what I am told	**0.514**	0.114
CDI16	I sleep pretty well	0.194	**0.480**
CDI19	I don't worry about aches and pains	-0.255	**0.332**
CDI07	I like myself	0.260	**0.560**
CDI15	Doing school work is not a big problem	0.040	**0.406**
CDI23	My school work is alright	0.161	**0.494**
CDI12	I like being with people	0.135	**0.588**
CDI20	I do not feel alone	-0.126	**0.465**
CDI22	I have plenty of friends	0.043	**0.524**
	*Number of items*	*19*	*8*
	*Cronbach’s alpha*	*0*.*87*	*0*.*55*
	*Polychromic alpha*	*0*.*91*	*0*.*63*
	*Overall Reliability*	*0*.*84(0*.*89)*	

Extraction Method: Principal Component Analysis.

### Factor analysis and item loadings

An EFA carried out on the first sample yielded a two-factor structure (Table 4). Nineteen of the 27 items of the CDI loaded on the first factor while eight loaded on the second factor. Items loaded on the first factor included sadness, crying always, being sure that terrible things will happen to one, fighting always and not feeling like eating. Generally, these items related to negative affect. Items loaded on factor 1 included being sure terrible things will happen to one had the highest factor loading (0.61), while never having fun at school had the lowest factor loading (0.40). On the other hand, items loading on the second factor relate to positive affect and include sleeping well, being alright with school work, liking oneself, etc. (Table 4). The results of the Horn’s parallel analysis (Table 5) and its associated scree plot (Fig 1) also suggested a 2-factor model for CDI in the present sample.

**Table 5:** Results of Horn's parallel analysis for component retention (for the Child Depression Inventory).

Component	Adjusted Eigenvalue	Unadjusted Eigenvalue	Estimated Bias
1	5.669760	5.984740	0.314979
2	1.519332	1.789761	0.270429

Adjusted eigenvalues > 1 indicate dimensions to retain. (2 components retained).

### Confirmatory factor analysis

**Fig 2:**
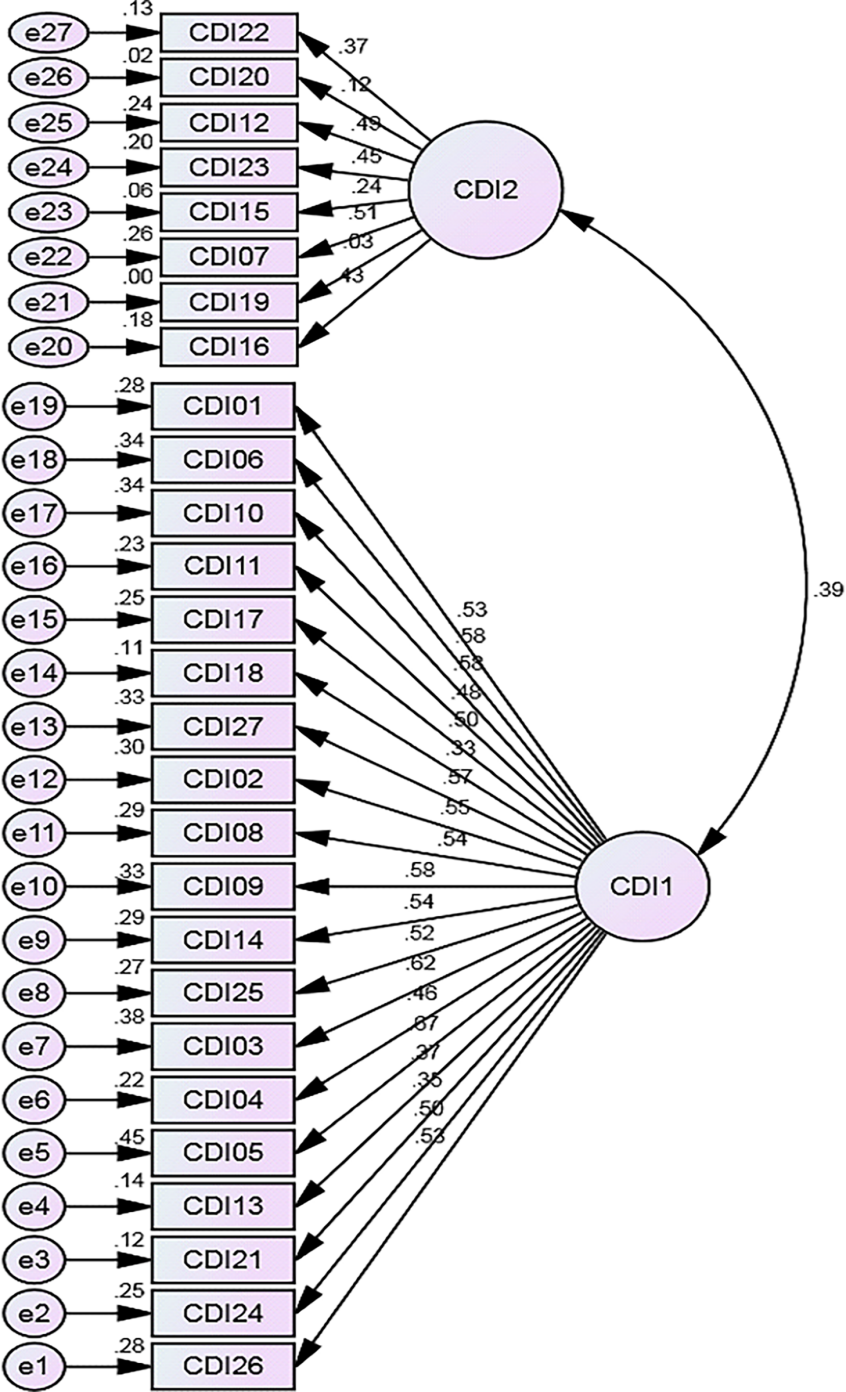
Path diagram showing the 2 factor model of the child depression inventory (hypothetical model).

Model Fit indices for the hypothesized and theoretical models are respectively shown in Table 6. Estimate of the minimum sample discrepancy (CMIN) and CMIN, divided by its degrees of freedom (CMIN/DF) was lower for the hypothesized model (CMIN = 928.00 and CMIN/DF = 2.87) than the theoretical three-factor model (CMIN = 117980.59 and CMIN/DF = 242.98), five-factor model (CMIN = 937.27 and CMIN/DF = 2.99) and six-factor model (CMIN = 126.72 and CMIN/DF = 5.24). Similarly, both the RMSEA and RMR were lower for the hypothesized two-factor model than all the theoretical models assessed while the values of the GFI, AGFI, PGFI, and CFI are higher for the 2-factor model. Apart from that, the value of the AIC was lower for the 2-factor model (1037.996) than the other models assessed in the present study. All Path coefficients of the 2-factor model (Fig 2) of the CDI (except two coefficients) were statistically significant and salient (>0.35) [28].

**Table 6:** Model fit indices of the theorectical and hypothesized models of the CDI.

Index	MODEL I	MODEL II	MODEL III	MODEL IV
CMIN	126.717	937.271	117980.590	927.996
CMIN/DF	5.243	2.985	242.981	2.873
RMR	0.022	0.036	0.189	0.022
GFI	0.878	0.921	-2.467	0.930
AGFI	0.822	0.905	-3.919	0.918
PGFI	0.605	0.765	-1.738	0.794
CFI	O.230	O.585	0.000	0.879
RMSEA	0.066	0.045	0.496	0.044
AIC	1486.717	1065.271	18042.590	1037.996
BIC	2019.793	1378.270	18194.199	1306.980

AIC- Akaike Information Criterion; CMIN- Minimum Sample discrepancy CMIN/DF-Ratio of Minimum sample discrepancy to its degree of freedom RMR- Root Mean Square Residual GFI- Goodnees of fit index; AGFI-Adjusted Goodnees of fit index; CFI- Comparative fit index; RMSEA-Root Mean Square Error of Approximation BIC- Baye’s Information Criterion. MODEL I–Six-Factor Model. MODEL II—Five-Factor Model. MODEL III–Three Factor Model. MODEL IV–Two-factor model extracted from the data used for the EFA.

## Discussion

The present study assessed the psychometric properties of the Child depression Inventory (CDI) in the population of apparently healthy adolescents in Benue state, Nigeria. Factor analysis was carried out to explore the underlying factor structure of the CDI in the Nigerian setting. The suggested factor structure was compared with existing factor structures of the CDI that has been reported in the literature [16, 23, 24, 31]. Though previous studies in Nigeria have used CDI to assess adolescent depression [3], it is crucial to assess the factor model that best describes the experiences of adolescents in this setting. Such exercise will not only provide data, but will also set the premise for an evidence-based use of the CDI in the setting. To the best of our knowledge, this study represents the first effort to examine the underlying factor structure of the CDI in a non-clinical sample in Nigeria.

Most studies using CDI to assess depression have focused on children with certain disease conditions but information on the factor structure of the CDI in community or epidemiologic studies is scanty in the literature [18, 28, 32]. For instance, the quasi-experimental study by Rivera et al. [18] was among a sample of Spanish adolescents who either met regular DSM-IV criteria, scored 13 or higher in the CDI or were deemed impaired by a clinical interviewer. Similarly, Nemets et al [32] carried out their study among children referred to psychiatric clinics of major hospitals in Israel. Given the diversity and severity of childhood and adolescent diseases, it is not entirely surprising that different underlying factor structures have been reported for the CDI; between two-factor and eight-factor structures have been reported in previous studies [23, 27]. These variations could be due to how the factors were determined, the type of participants, the method of factor extraction and rotation or other variations in the methodology [23]. Notwithstanding, Logan et al. [27] opined that these varying factor structures are indications that, across samples, the CDI may not be able to uniformly measure the experiences of childhood depression. The present study was not different, as we found a two-factor structure for the CDI. The items of the Child Depression Inventory were loaded on two factors against the original 5-factor model of the CDI [27, 33,34]Both the overall and sub-scale factor structures reported in this study showed high internal consistency as opposed to that of the original five factor model. This may further explain why the model fit analysis for the 2-factor model was better in the present study. This finding is corroborated by the results of similar studies [18, 27, 34]. In a study conducted among adolescents and children with chronic pain, Logan and colleagues [27] reported low internal consistencies for the subscales of the original factor structure of the CDI. The overall factor structure, however, showed high internal consistency. In the present study, for instance, the internal consistencies of the original five factor structure ranged from 0.36 to 0.62, which is quite lower than what was obtained in Logan et al [27], which had internal consistency values ranging from 0.54 to 0.71. Logan et al. [27] concluded that this may be a further indication that the original factor structure of the CDI, proposed by Kovacs [16], does not sufficiently explain the underlying factor structure in their sample. However, using a 26-item version of the CDI (upon dropping the suicide item), Cole and Martin [34], reported high internal consistencies ranging from 0.88 to 0.91.

This present study reports a two-factor model with items that relate to positive affect loading on the first factor while negative affect-related items loading on the second factor. This may be attributed to the fact that as against other studies, especially in this setting, this was done among apparently healthy cohort of adolescents. Previous work by Craighead et al. [24] corroborates this finding. Although their work reported a six-factor model (Externalizing, Dysphoria, Self-depreciation, School Problems, Social Problems and Biological Dysregulation), two distinct higher order factors (which they called Externalizing and Internalizing) were also obtained [23, 24]. These sub-scales both show relatively high internal consistency similar to those of the original CDI validation sample and studies reported by previous studies [27,34]. The two-factor model of the Child Depression inventory in the present analysis showed a good fit. The measures of model fit meet the criteria proposed [35–37] and which has been used by previous studies [28, 37]. Therefore, though our sample consisted mainly of adolescents aged 10–19 years, it is very likely that the suggested 2-factor model could be used among children as no item was modified from its original content and meaning as contained in the original CDI scale [16]. However, the validity of such application could be investigated in a future study.

This study has a few limitations. First, the present analysis studied apparently healthy adolescents and due to limitation of data, we did not control for the validity and sensitivity of the new model been recommended. However, a future efforts on the recommended model may possibly focus on this direction. Also, the present study is a secondary analysis of data based on a cross–sectional study which by design may be affected by selection biases but the robust sample size and the analysis provided in the present study are some of the obvious strengths of the analyses. Moreover, the two-factor model presented here has many advantages including being easy to score, high reliability estimates (as obtained in the present analyses), good factor loading and fit indices which is an indication of the stability of the factors.

In conclusion, the CDI is a viable tool for assessing childhood and adolescent depression with wide application. However, an assessment of depression in the current setting yielded a two factor structure of the CDI with factors relating to optimistic or positive view of self or life (with positive affect items) and pessimistic or negative view of self or life (with negative affect items).
